# Anti-Inflammatory and Antioxidant Effects of *Carpesium cernuum* L. Methanolic Extract in LPS-Stimulated RAW 264.7 Macrophages

**DOI:** 10.1155/2020/3164239

**Published:** 2020-08-07

**Authors:** Yea-Jin Park, Se-Yun Cheon, Dong-Sung Lee, Divina C. Cominguez, Zhiyun Zhang, Sangwoo Lee, Hyo-Jin An

**Affiliations:** ^1^Department of Pharmacology, College of Korean Medicine, Sangji University, Wonju, Gangwon-do 26339, Republic of Korea; ^2^Department of Korean Medical Science, School of Korean Medicine and Healthy Aging Korean Medical Research Center, Pusan National University, Yangsan, Gyeongnam, 50612, Republic of Korea; ^3^College of Pharmacy, Chosun University, 309 Pilmun-daero, Dong-gu Gwangju 61452, Republic of Korea; ^4^State Key Laboratory of Systematic and Evolutionary Botany, Institute of Botany, The Chinese Academy of Sciences, Beijing 100093, China; ^5^International Biological Material Research Center, Korea Research Institute of Bioscience and Biotechnology, Daejeon 34141, Republic of Korea

## Abstract

A hypernomic reaction or an abnormal inflammatory process could cause a series of diseases, such as cardiovascular disease, neurodegeneration, and cancer. Additionally, oxidative stress has been identified to induce severe tissue injury and inflammation. *Carpesium cernuum* L. (C. *cernuum*) is a Chinese folk medicine used for its anti-inflammatory, analgesic, and detoxifying properties. However, the underlying molecular mechanism of C. *cernuum* in inflammatory and oxidative stress conditions remains largely unknown. The aim of this study was to examine the effects of a methanolic extract of C. *cernuum* (CLME) on lipopolysaccharide- (LPS-) induced RAW 264.7 mouse macrophages and a sepsis mouse model. The data presented in this study indicated that CLME inhibited LPS-induced production of proinflammatory mediators such as nitric oxide (NO) and prostaglandin E_2_ (PGE_2_) in RAW 264.7 cells. CLME treatment also reduced reactive oxygen species (ROS) generation and enhanced the expression of heme oxygenase-1 (HO-1) protein in a dose-dependent manner in the LPS-stimulated RAW 264.7 cells. Moreover, CLME treatment abolished the nuclear translocation of nuclear factor-*κ*B (NF-*κ*B), enhanced the activation of nuclear factor-erythroid 2 p45-related factor 2 (Nrf2), and reduced the expression of extracellular signal-related kinase (ERK) and ERK kinase (MEK) phosphorylation in LPS-stimulated RAW 264.7 cells. These outcomes implied that CLME could be a potential antioxidant and anti-inflammatory agent.

## 1. Introduction

Inflammation is a multistep process occurring in many animals. It is also one of the first lines of defense against harmful stimuli, such as trauma, bacteria, and irritants [1]. Acute inflammation is a finite process, leading to the return of tissue homeostasis. However, it has been reported that unrestrained inflammation occurs during chronic inflammatory milieu, such as allergies, asthma, arthritis, atherosclerosis, multiple sclerosis, metabolic syndromes, and obesity [2, 3]. Hence, controlling unbalanced inflammation is a potentially crucial strategy for preventing inflammatory environments. Macrophages detect and react to some pathogens through pattern-recognition receptors (PRRs) containing toll-like receptors (TLRs) and consequently regulate the inflammatory response [4]. Lipopolysaccharide (LPS), a well-known inflammatory ligand, can activate macrophages to release a variety of inflammatory cytokines [5]. Therefore, we can mimic inflammatory and oxidative stress milieu using RAW 264.7 macrophage cells, which demonstrate a highly reproducible response to LPS.

LPS stimulates TLR4 [6], and the LPS-initiated signaling cascade results in the activation of mitogen-activating protein kinase (MAPK) and the nuclear factor-*κ*B (NF-*κ*B) inflammatory signaling pathway [7]. Moreover, activated macrophages result in the secretion of many proinflammatory molecules including reactive oxygen species (ROS), nitric oxide (NO), inducible nitric oxide synthase (iNOS), and prostaglandin E_2_ (PGE_2_) that have been known to be important components in tissue destruction and the associated pathological process [8, 9].

Oxidative stress is an important inducer for inflammation [10]. High levels of ROS can induce a considerable decrease in the antioxidant defense mechanisms resulting in DNA, protein, and lipid damage [11]. Furthermore, ROS modulate the intracellular reduction-oxidation- (redox-) sensitive signaling and nuclear transcription factors including NF-*κ*B and nuclear factor-erythroid 2 p45-related factor 2 (Nrf2) in LPS-challenged macrophage activation [12]. Nrf2 signaling constitutes one of the most prominent cellular defense mechanisms in xenobiotic damage and oxidative stress [13] and is ubiquitously expressed in extensive cell types and tissues [10]. In addition, Nrf2 is a chief transcription factor regulating heme oxygenase-1 (HO-1) and displays its effects by translocating to the nucleus [14]. HO-1 is an antioxidant enzyme induced by oxidative stress; however, overexpression of HO-1 prior to stimulation with LPS remarkably declined the production of subsequent inflammatory mediators such as NO, interleukin-6 (IL-6), and monocyte chemoattractant protein-1 (MCP-1) [15]. In this respect, antioxidants are the first line of defense against oxidative damage induced by scavenging radicals and thus act to maintain optimal health [16].

*Carpesium cernuum* L. (C. *cernuum*), drooping carpesium, is a member of Compositae, and this is a Chinese folk medicine used for eliminating heat and toxic material, detumescence, and relieving pain [17]. More precisely, C. *cernuum* has been used to treat tonsillitis, mumps, dysentery, toothache, and mastitis in China [18]. Furthermore, some compounds isolated from C. *cernuum* were reported to possess antiplasmodial activity against *Plasmodium falciparum* and *Plasmodium berghei* in mice [19]. However, no report on the underlying molecular mechanism of C. *cernuum* in inflammation and oxidative stress has been recorded. Accordingly, this current study was designed to investigate the effects of methanolic extract of C. *cernuum* (CLME) on LPS-induced inflammation and oxidative stress via targeting the expression of NF-*κ*B, Nrf2/Kelch-like ECH-associated protein 1 (Keap1), and extracellular signal-regulated kinase (ERK) in RAW 264.7 macrophages. Furthermore, we also examined the impact of CLME on an LPS-induced sepsis mouse model.

## 2. Materials and Methods

### 2.1. Chemicals and Reagents

CLME were obtained from the International Biological Material Research Center (Daejeon, Republic of Korea). Dulbecco's modified Eagle's medium (DMEM), fetal bovine serum (FBS), penicillin, and streptomycin were purchased from Life Technologies Inc. (Grand Island, NY, USA). LPS (*Escherichia coli*, serotype 0111:B4), 3-(4,5-dimethylthiazol-2-yl)-2,5-diphenyltetrazolium bromide (MTT), N^6^-(1-Iminoethyl)-L-lysine (NIL), NS-398, Griess reagent, and (1R)-(-)-Myrtenal (≥95%) were purchased from Sigma-Aldrich (St. Louis, MO, USA). Dimethyl sulfoxide (DMSO) was purchased from Junsei Chemical Co., Ltd. (Tokyo, Japan). The inhibitor of *κ*B (I*κ*B) (cat. No. sc-203), phospho- (p-) I*κ*B (cat. No. sc-8404), p65 (cat. No. sc-109), Keap1 (cat. No. sc-514914), HO-1 (cat. No. sc-136960), ERK kinase (MEK) (cat. No. sc-81504), p-MEK (cat. No. sc-81503), *α*-tubulin (cat. No. sc-8035), and *β*-actin (cat. No. sc-47778) monoclonal antibodies, as well as the peroxidase-conjugated secondary antibody, were purchased from Santa Cruz Biotechnology, Inc. (Santa Cruz, CA, USA). iNOS (cat. No. #2982), Nrf2 (cat. No. #12721), ERK 1/2 (cat. No. #9102), p-ERK 1/2 (cat. No. #9101), p38 (cat. No. #9212), p-38 (cat. No. #9211), c-Jun N-terminal kinase (JNK) (cat. No. #9252), p-JNK (cat. No. #9251), and poly-adenosine diphosphate (ADP) ribose polymerase (PARP) (cat. No. #9542) antibodies were purchased from Cell Signaling Technology (Danbers, MA, USA). The enzyme immunoassay kits for PGE_2_, tumor necrosis factor-*α* (TNF-*α*), and IL-6 were obtained from R&D Systems (Minneapolis, MN, USA). MUSE® oxidative stress assay kit for ROS was obtained from Merck (Darmstadt, Germany).

### 2.2. Preparation of CLME

CLME was obtained from the International Biological Material Research Center (Daejeon, Republic of Korea). More precisely, C. *cernuum* (8BF2C268-9F3D-4496-ADD8-9F3ABCBC5590) was collected from the Zheshang Park, Wuhu City, Anhui Province, in China and was identified by Dr. Zhiyun Zhang at the Herbarium, Institute of Botany, The Chinese Academy of Sciences in 2012. A voucher specimen (accession number KRIB 0050072) of the retained material is preserved at the herbarium of KRIBB. The dried and refined whole plant including roots of C. *cernuum* (32 g) was extracted with 1 L of 99.9% (*v*/*v*) methanol, with repeated sonication (15 min) and resting (2 h) for 3 days at 45°C. The resultant product was filtered with nonfluorescence cottons and concentrated using the rotary evaporator (N-1000SWD, EYELA) under reduced pressure at 45°C. Finally, a total of 3.52 g of CLME was obtained by freeze-drying and the yield was calculated at 11%.

### 2.3. Animal Experiments of Septic Shock Model

Six-week-old male C57BL/6N mice were purchased from Daehan Biolink Co. (Daejeon, Republic of Korea). Prior to these experiments, the Institutional Animal Care and Use Committee (IACUC) of Sangji University approved all the experimental protocols (approved number 2019-18). Mice were maintained at 22 ± 2°C and 55 ± 9% humidity, under a 12 h light/dark cycle, and provided water and diet ad libitum. Mice were divided into three groups (*n* = 5 per group) as follows: LPS-treated group (LPS) and LPS+oral administration of CLME groups (CLME 50 or 100 mg/kg). LPS was dissolved in phosphate-buffered saline (PBS), and CLME was dissolved in 18 : 1 : 1 ratio of distilled water, ethanol, and cremophor (vehicle). The mice were orally administered either vehicle alone or vehicle with CLME for 1 h before LPS injection (25 mg/kg). The survival was recorded at different intervals.

### 2.4. Cell Culture

The RAW 264.7 cell line was obtained from the Korea Cell Line Bank (KCLB, Seoul, Republic of Korea). The cells were cultured in DMEM supplemented with 10% FBS, penicillin (100 U/mL), and streptomycin (100 *μ*g/mL) at 37°C and a humidified atmosphere of 5% CO_2_. The CLME was dissolved in DMSO and filtered using Acrodisc® Syringe Filters 0.2 *μ*m Supor® Membrane (Rall Life Sciences, MI, USA).

### 2.5. MTT Assay for Cell Viability

Cell viability was assessed using the MTT assay. Briefly, RAW 264.7 cells were seeded into a 96-well plate at a density of 1 × 10^4^ cells per well and then treated with various concentrations of CLME for 24 h. After treatment, CLME-treated cells were incubated with the MTT solution (5 mg/mL) for 4 h at 37°C. After discarding the supernatant, the formazan product that formed in the cell was dissolved in DMSO and measured at 570 nm using an Epoch microplate spectrometer (BioTek, Winooski, VT, USA).

### 2.6. NO Assay

NO content was analyzed indirectly by measuring the supernatants of cultured RAW 264.7 cells for nitrite using the Griess reagent (1% sulfanilamide in 5% phosphoric acid, 1% *α*-naphthylamide in H_2_O). RAW 264.7 cells were seeded into a 24-well plate at a density of 5 × 10^5^ cells per well and then treated with various concentrations of CLME for 1 h. NIL was used as a positive iNOS selective inhibitor. After preincubation with CLME and NIL, the cells were stimulated with LPS (1 *μ*g/mL) for 48 h. Cell culture media (50 *μ*L) was mixed with 50 *μ*L of Griess reagent in a 96-well plate, incubated at room temperature for 15 min, and then measured at 540 nm using an Epoch microplate spectrometer (BioTek, Winooski, VT, USA).

### 2.7. PGE_2_ Assay

RAW 264.7 cells were seeded into a 24-well plate at a density of 5 × 10^5^ cells per well and then treated with various concentrations of CLME for 1 h. NS-398 was used as a positive cyclooxygenase-2 (COX-2) selective inhibitor. After preincubation with CLME and NS-398, the cells were stimulated with LPS (1 *μ*g/mL) for 24 h. The release of PGE_2_ in the cultured media was measured using the enzyme-linked immunosorbent assay (ELISA) kit according to the manufacturer's instructions.

### 2.8. ROS Assay

The cells were prepared in a 60 mm dish at a density of 1 × 10^6^ cells and then treated with various concentrations of CLME for 1 h. After preincubation with CLME, the cells were activated with LPS (1 *μ*g/mL) for 1 h and mixed with Muse® Oxidative Stress Reagent working solution. The cells were incubated for 30 min at 37°C and detected using the Muse® Cell Analyzer (Merck, Darmstadt, Germany).

### 2.9. Preparation of the Nuclear and Cytosolic Extract

The cells were plated in 60 mm dishes (1 × 10^6^ cells/mL) and treated with CLME for 1 h. And then, cells were stimulated with LPS (1 *μ*g/mL) to check the expression of NF-*κ*B and Nrf2 for 30 min and 1 h, respectively. For cytosolic extraction, the hypotonic buffer (10 mM HEPES (pH 7.9), 1.5 mM MgCl_2_, 10 mM KCl, 0.2 mM phenylmethylsulfonyl fluoride (PMSF), 0.5 mM dithiothreitol (DTT), and 10 mg/mL aprotinin) was added to the cell. The cells are harvested by scraping with the hypotonic buffer and incubated on ice for 20 min. The cells were then lysed by adding 0.1% Nonidet P-40, vortexed vigorously for 10 sec, and centrifuged at 12,000 *×* g for 5 min. The supernatant was used for cytosolic extracts. For nuclear extraction, the pellet was resuspended in a high salt buffer (20 mM HEPES (pH 7.9), 25% glycerol, 400 mM KCl, 1.5 mM MgCl_2_, 0.2 mM ethylenediaminetetraacetic acid (EDTA), 0.5 mM DTT, 1 mM NaF, and 1 mM sodium orthovanadate), vortexed for 30 min at 4°C, and centrifuged at 12,000 *×* g for 15 min. The resulting supernatant was sued for nuclear extracts.

### 2.10. Western Blot Analysis

The cells were plated in 60 mm dishes (2 × 10^5^ cells/mL) and treated with CLME. After 1 h, the LPS (1 *μ*g/mL) was treated to the cells in order to examine the expression of p-MEK for 20 min, I*κ*B, p-I*κ*B, and MAPKs for 30 min and iNOS for 24 h. First, cells were suspended in PRO-PREP™ protein extraction solution (Intron Biotechnology, Seoul, Republic of Korea). The suspension was incubated on ice for 20 min and then centrifuged at 11,000 × g for 30 min. The protein concentration was determined using the Bio-Rad protein assay reagent according to the manufacturer's instructions (Bio-Rad, Hercules, CA, USA). Equal amounts (20 *μ*g) of the protein samples were separated on a sodium dodecyl sulfate (SDS) polyacrylamide gel, followed by transfer onto a polyvinylidene fluoride (PVDF) membrane. Membranes were incubated for 30 min with 2.5% skim milk at 20-25°C and then incubated overnight with a 1 : 1000 dilution of the primary antibody at 4°C. The blots were washed three times with Tween 20/Tris-buffered saline (T/TBS) and incubated with a 1 : 2500 dilution of horseradish peroxidase-conjugated secondary antibody for 2 h at 20-25°C. The blots were further washed three times with T/TBS and finally visualized using enhanced chemiluminescence (GE Healthcare, Waukesha, WI, USA).

### 2.11. HPLC Analysis

HPLC was performed to identify the component profile of CLME. The extract was precisely quantified (5 mg), dissolved in 1 mL of MeOH, and filtered to prepare an extract sample. The HPLC and ODS-A HPLC column (YMC-pack ODS-A, 250 × 4.6 mm I.D., S-5 *μ*m, 12 nm, YMC, Kyoto, Japan), consisting of a pump (1525 Binary HPLC pump, Waters, MA, USA) and a PDA detector (996 Photodiode array detector, waters, MA, USA), were connected for analysis. As mobile solvent systems, A channel: 0.1% acetic acid distilled water, and B channel: 0.1% acetic acid acetonitrile, were used. The slope profile proceeded as follows: 0-5 min, 5-10% B linear; 5-15 min, 10% B linear; 15-20 min, 10-20% B linear; 20-40 min, 20% B linear; 40-60 min, 20-70% B linear; and 60-70 min, 70-100% B. The flow rate was maintained at 1.0 mL/min during the analysis. The CLME solution (20 *μ*L) was injected at 5 mg/mL, and 20 *μ*L of 25 *μ*g/mL of (1R)-(-)-Myrtenal (≥95%, Sigma-Aldrich, St. Louis, MO, USA) dissolved in MeOH was injected as a standard sample. The detection wavelength was adjusted to 252 nm. The different concentrations of (1R)-(-)-Myrtenal (1.5625, 3.125, 6.25, 12.5, 25, and 50 *μ*g/mL) were subjected to quantitative analysis using the calibration graph.

### 2.12. Cytokine Assay

The cells were transferred into 24-well dishes at a density of 1 × 10^5^ cells and then treated with various concentrations of CLME for 1 h. Next, the cells were activated with LPS (1 *μ*g/mL) for 24 h, and the culture media were collected and stored at -80°C. The levels of TNF-*α* and IL-6 were measured using ELISA kits according to the manufacturer's instructions.

### 2.13. Statistical Analysis

Each result is expressed as the mean ± standard deviation (S.D.) of triplicate experiments. Statistical analysis was performed using SPSS statistical analysis software (version 19.0; International Business Machines, Armonk, NY, USA). Statistically significant differences were determined using analysis of variance and Dunnett's post hoc test, and *P* values of less than 0.05 were considered statistically significant.

## 3. Results

### 3.1. Effect of CLME on Survival Rate in LPS-Induced Septic Shock Mouse Model and Production of NO and PGE_2_ and Expression of iNOS Protein in LPS-Stimulated RAW 264.7 Cells

**Figure 1:**
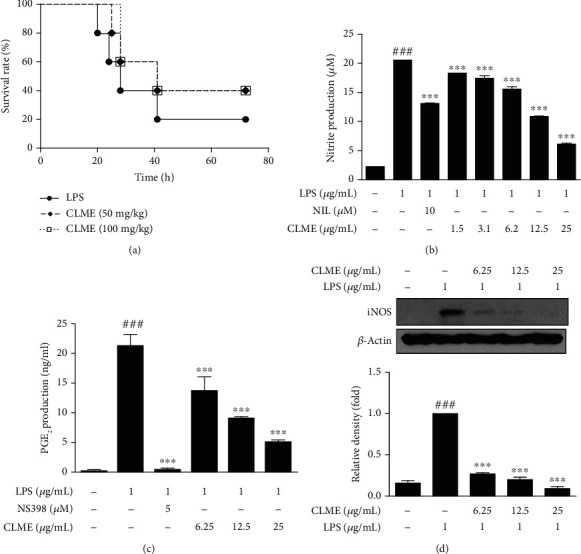
CLME prevents septic death in an LPS-induced endotoxemia mouse model and inhibits the levels of NO, PGE_2_, and iNOS in LPS-stimulated RAW 264.7 cells. (a) The graph represents survival rate after LPS injection (25 mg/kg) with or without CLME treatment (50 or 100 mg/kg). RAW 264.7 cells were pretreated with indicated concentrations of CLME for 1 h and followed by LPS stimulation (1 *μ*g/mL) for indicated times. (b) NO production was analyzed indirectly by measuring the supernatants of cultured RAW 264.7 cells for nitrite using the Griess reagent. (c) The prostaglandin E_2_ (PGE_2_) production in the culture media was measured by ELISA kit. (d) The iNOS expression was analyzed by western blot analysis. Densitometric analysis was performed using ImageJ ver. 1.50i. The values are represented as the mean ± S.D. (*n* = 4). ^###^*P* < 0.001 vs. the CON group; ^∗∗∗^*P* < 0.001 vs. the LPS-treated group.

To examine the anti-inflammatory effect of CLME *in vivo*, mice were pretreated with CLME (50 or 100 mg/kg) for 1 h and then injected with LPS (25 mg/kg). The survival was monitored every 10-12 h for 3 days. In the absence of CLME, 60% mice died within 28 h of LPS injection, and totally, 80% mice died within 41 h. On the other hand, only 60% of septic mice with CLME-treated groups were dead within 41 h of LPS injection, indicating that CLME improved the survival of mice suffering from lethal endotoxic shock (Figure 1(a)). Prior to the *in vitro* antioxidant and anti-inflammatory evaluation, we initially examined the effect of CLME on cytotoxicity in RAW 264.7 cells at the indicated concentrations (15.63, 31.25, 62.5, 125, 250, and 500 *μ*g/mL) using the MTT assay. As shown in Fig. S1A, the cell viability was unaffected by CLME at concentrations up to 31.25 *μ*g/mL, whereas higher concentrations of CLME showed obvious cytotoxicity. Therefore, concentrations of CLME less than 31.25 *μ*g/mL (6.25, 12.5, and 25 *μ*g/mL) were chosen for the subsequent experiments. Furthermore, RAW 264.7 cell morphology was also observed under an optical microscope. The protective effect of CLME was revealed in the morphological changes induced by LPS stimulation. In cells pretreated with CLME, the level of cell spreading and pseudopodia induced by LPS was decreased, indicating inhibition of cell activation or differentiation (Fig. S1B). Redundant NO and PGE_2_, as inflammatory mediators, have been regarded as the main causes for acute inflammatory responses as well as chronic inflammatory diseases, such as osteoarthritis, rheumatoid arthritis, and inflammatory bowel disease [20]. In order to examine the effect of CLME on level of inflammatory mediators, we investigated NO and PGE_2_ production in LPS-stimulated RAW 264.7 cells. The cells were treated with or without CLME for 1 h and then treated with LPS (1 *μ*g/mL). CLME treatment significantly (*P* < 0.001) suppressed LPS-induced NO production in a dose-dependent manner. In particular, high concentration of CLME (25 *μ*g/mL) demonstrated elevated inhibitory effects on NO production than NIL, used as a positive iNOS selective inhibitor (Figure 1(b)). Moreover, LPS-induced PGE_2_ production was significantly (*P* < 0.001) decreased by CLME treatment in a dose-dependent manner, and NS-398 was also used as a positive COX-2 selective inhibitor (Figure 1(c)). The expression of the iNOS protein was increased after stimulation with LPS in RAW 264.7 cells, and we determined whether CLME could repress iNOS protein expression. As demonstrated by western blot analysis, cells pretreated with CLME strongly (*P* < 0.001) repressed the expression of the iNOS protein in a dose-dependent manner (Figure 1(d)). As a result, it can be established that CLME possesses anti-inflammatory ability through the mitigation of the proinflammatory enzyme, iNOS, and the mediators, NO and PGE_2_.

### 3.2. Effect of CLME on NF-*κ*B Activation in LPS-Stimulated RAW 264.7 Cells

**Figure 2:**
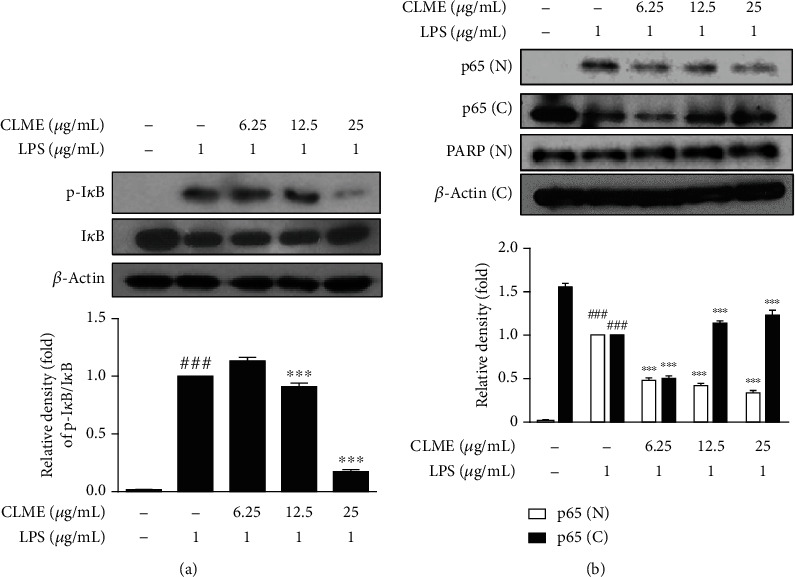
CLME inhibits the phosphorylation and degradation of I*κ*B and nuclear translocation of NF-*κ*B in LPS-stimulated RAW 264.7 cells. RAW 264.7 cells were pretreated with indicated concentrations of CLME for 1 h and followed by LPS stimulation (1 *μ*g/mL) for 30 min. (a) The I*κ*B, p-I*κ*B, and (b) p65 expressions were analyzed by western blot analysis. Densitometric analysis was performed using ImageJ ver. 1.50i. The values are represented as the mean ± S.D. (*n* = 3). ^###^*P* < 0.001 vs. the CON group; ^∗∗∗^*P* < 0.001 vs. the LPS-treated group.

To establish the cellular mechanism involved in the anti-inflammatory activity of CLME-related inhibition of NO, PGE_2_, and iNOS levels, the protein expressions of I*κ*B, p-I*κ*B, and NF-*κ*B were detected in the LPS-stimulated RAW 264.7 cells. As shown in Figure 2(a), a difference in I*κ*B-*α* expression was noted between the CON and LPS-treated groups. The CLME treatment (25 *μ*g/mL) had notably higher I*κ*B expression than the LPS-treated group. Moreover, LPS treatment remarkably increased the phosphorylation of I*κ*B compared to the CON group. Conversely, high concentration of CLME reported markedly (*P* < 0.001) lower phosphorylation of I*κ*B compared to the LPS-treated group. There was also a significant difference in p65 expression between the CON group and the LPS group, and the CLME-treated groups demonstrated significantly lower nuclear p65 expression than the LPS-treated group (*P* < 0.001). Additionally, the CLME-treated group (25 *μ*g/mL) showed notably (*P* < 0.001) higher cytosolic p65 expression than the LPS-treated group. Overall, the nuclear translocation of NF-*κ*B was inhibited by CLME in a dose-dependent manner (Figure 2(b)). Notably, the suppression of NF-*κ*B protein exhibited a similar pattern to the inhibition of NO, PGE_2_, and iNOS levels.

### 3.3. Effect of CLME on ROS Generation in LPS-Stimulated RAW 264.7 Cells

**Figure 3:**
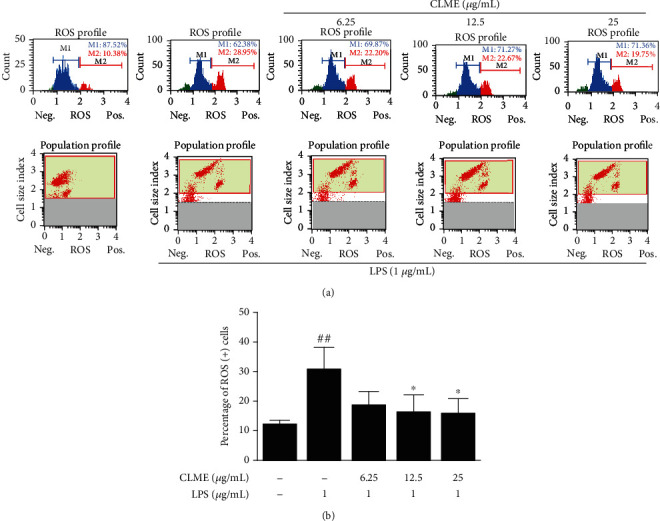
CLME inhibits the generation of ROS in LPS-stimulated RAW 264.7 cells. RAW 264.7 cells were pretreated with indicated concentrations of CLME for 1 h and followed by LPS stimulation (1 *μ*g/mL) for 1 h. (a) The plot shows the histogram of gated cells with two markers providing data on two cell populations: ROS neg. (-) and ROS pos. (+) cells, indicating M1 and M2, respectively. ROS generations were analyzed by MUSE® oxidative stress assay kit. (b) The graph for percentage of ROS pos. (+) cells. The values are represented as the mean ± S.D. (*n* = 3). ^##^*P* < 0.01 vs. the CON group; ^∗^*P* < 0.05 vs. the LPS-treated group.

The elevated levels of ROS through modulation of signaling pathways boosts the generation of a proinflammatory condition and sustain disease progression [21]. To identify whether CLME has direct antioxidant potential in LPS-stimulated RAW 264.7 cells, the cellular ROS scavenging activity was assessed using the MUSE® oxidative stress assay. ROS neg. (negative) and ROS pos. (positive) cells indicate M1 and M2, respectively. As shown in Figure 3, the LPS-treated group provoked a remarkable increase (28.95%) in intracellular ROS generation compared to the CON group (10.38%), whereas the CLME-treated groups resulted in 22.20, 22.67, and 19.75% on the ROS generation ratio in LPS-stimulated RAW 264.7 cells. Based on these findings, we demonstrated that CLME possesses antioxidant capacity by scavenging the generated ROS.

### 3.4. Effect of CLME on the Expression of HO-1 and Nrf2/Keap1 in LPS-Stimulated RAW 264.7 Cells

**Figure 4:**
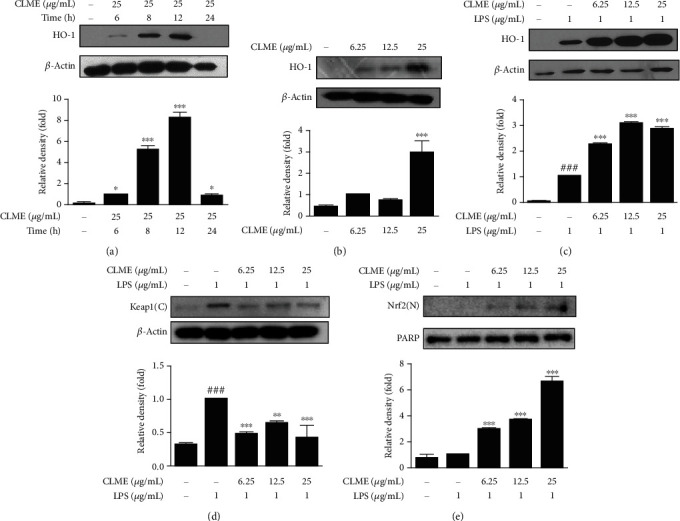
CLME regulates the expression of HO-1 and Keap1/Nrf2 in LPS-stimulated RAW 264.7 cells. (a–c) HO-1 expression in RAW 264.7 cells. (a) The cells were treated with CLME of 25 *μ*g/mL for the indicated times. (b) The cells were treated with CLME of indicated concentrations for 12 h. The values are represented as the mean ± S.D. (*n* = 3). ^∗^*P* < 0.05 and ^∗∗∗^*P* < 0.001 vs. the CON group. (c) The cells were pretreated with indicated concentrations of CLME for 1 h and followed by LPS stimulation (1 *μ*g/mL) for 12 h. (d, e) The cells were pretreated with indicated concentrations of CLME for 1 h and followed by LPS stimulation for 1 h. (d) Keap1 and (e) Nrf2 expressions were analyzed by western blot analysis. Densitometric analysis was performed using ImageJ ver. 1.50i. The values are represented as the mean ± S.D. (*n* = 3). ^###^*P* < 0.001 vs. the CON group; ^∗∗^*P* < 0.01 and ^∗∗∗^*P* < 0.001 vs. the LPS-treated group.

To clarify the impact of CLME on the expression of HO-1, a cellular antioxidant enzyme, RAW 264.7 cells were treated with LPS for 4, 6, 8, 12, and 24 h. The expression of HO-1 was increased by LPS treatment in RAW 264.7 cells, peaking at 12 h (Fig. S2A). In addition, the time-dependent western blot analysis indicated greatly (*P* < 0.001) increased protein expression of HO-1, from 6 to 12 h, peaking at 12 h after CLME treatment (Figure 4(a)). Furthermore, the expression of HO-1 was also increased and maintained by CLME treatment during 12 h in LPS-stimulated RAW 264.7 cells (Fig. S2B). Based on the above results, we further confirmed the expression of HO-1 at 12 h with three concentrations of CLME (6.25, 12.5, and 25 *μ*g/mL). The data demonstrated that CLME gradually (*P* < 0.001) enhanced the expression of HO-1 in a dose-dependent manner in RAW 264.7 cells treated with or without LPS (Figures 4(b) and 4(c)). These results demonstrated that CLME possesses antioxidant activity by regulating ROS generation and HO-1 expression. Oxidative stressors and some anti-inflammatory electrophilic drugs interrupt Keap1-Nrf2 complex, promote the nuclear translocation of Nrf2, and finally activate the transcription of antioxidant genes including HO-1 [22]. Therefore, we hypothesized that the regulatory effect of CLME on LPS-induced oxidative stress (e.g., intracellular ROS generation) and HO-1 expression may be controlled by the Keap1/Nrf2 signaling pathway. LPS treatment increased the cytosolic expression of the Keap1 protein. Conversely, CLME treatment dramatically (*P* < 0.001) attenuated LPS-induced cytosolic Keap1 expression (Figure 4(d)). Moreover, it was also noted that CLME treatment resulted in a strong (*P* < 0.001) upregulation in the nuclear accumulation of Nrf2 expression compared to the LPS group (Figure 4(e)). These results postulated that CLME activates the Keap1/Nrf2 signaling, shedding light on the molecular mechanism underlying its antioxidant effects.

### 3.5. Effect of CLME on MEK/ERK Activation in LPS-Stimulated RAW 264.7 Cells

**Figure 5:**
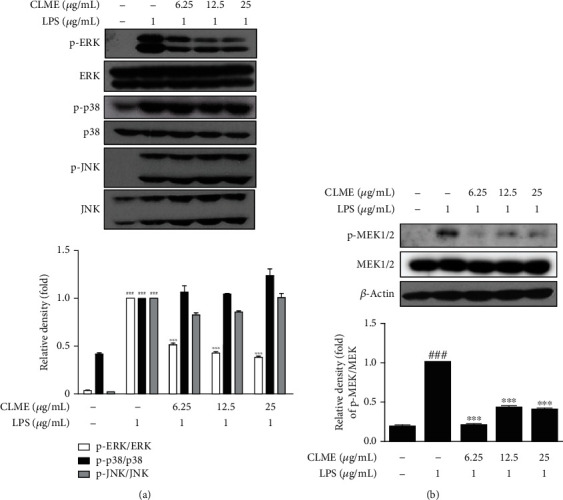
CLME inhibits the phosphorylation of MEK/ERK in LPS-stimulated RAW 264.7 cells. RAW 264.7 cells were pretreated with indicated concentrations of CLME for 1 h and followed by LPS stimulation (1 *μ*g/mL) for (a) 30 min and (b) 20 min. These MAPK and MEK protein expressions were analyzed by western blot. Densitometric analysis was performed using ImageJ ver. 1.50i. The values are represented as the mean ± S.D. (*n* = 3). ^###^*P* < 0.001 vs. the CON group; ^∗∗∗^*P* < 0.001 vs. the LPS-treated group.

To verify the hypothesis that CLME predominantly signals through the MAPK pathway in the context of NF-*κ*B inactivation and Nrf2 activation, we evaluated the effect of CLME on the MAPK signaling pathway in LPS-stimulated RAW 264.7 cells. As shown in Figure 5(a), the activation of MAPK (p38, ERK, and JNK) signaling occurred in the LPS-treated group compared to the CON group. CLME significantly (*P* < 0.001) declined the phosphorylation of ERK compared to the LPS group. However, the expressions of phosphorylated JNK and p38 MAPKs were unaffected by CLME. In addition, the impact of CLME on the expression of MEK, which is upstream of ERK, was evaluated, and CLME treatment effectively (*P* < 0.001) alleviated the expression of p-MEK compared to the LPS-treated group (Figure 5(b)).

### 3.6. Anti-Inflammatory Effect of Myrtenal as a Compound of CLME

**Figure 6:**
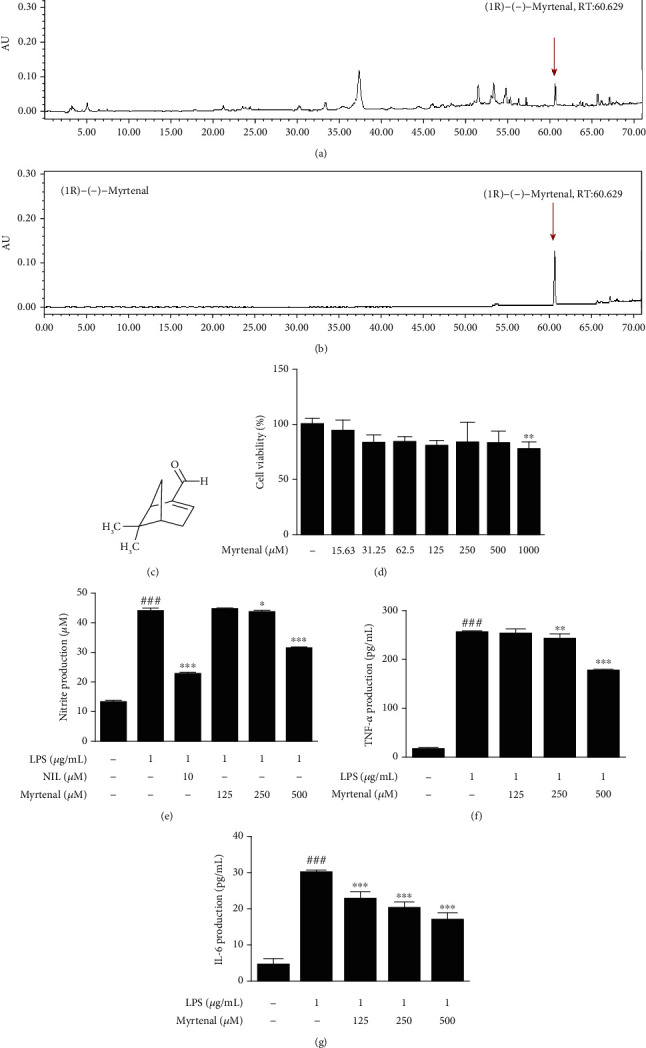
Myrtenal inhibits the production of NO, TNF-*α*, and IL-6 in LPS-stimulated RAW 264.7 cells. HPLC chromatogram of CLME (a) and (1R)-(-)-Myrtenal (b) as a standard compound. (c) Chemical structures of Myrtenal. (d) The viability of Myrtenal in RAW 264.7 cells. The values are represented as the mean ± S.D. (*n* = 6). ^∗∗^*P* < 0.01 vs. the CON group. (e) The nitrite production was determined using Griess reagent. (f) TNF-*α* and (g) IL-6 production in the culture media was measured by ELISA kit. The values are represented as the mean ± S.D. (*n* = 3). ^###^*P* < 0.001 vs. the CON group; ^∗^*P* < 0.05, ^∗∗^*P* < 0.01, and ^∗∗∗^*P* < 0.001 vs. the LPS-treated group.

We have confirmed the HPLC chromatogram of C. cernuum extract and the standard compound (1R)-(-)-Myrtenal. As shown in Figure 6(a), the retention time of the main peak in CLME was 60.629 min. In addition, it was confirmed that the peak of (1R)-(-)-Myrtenal appeared at 60.629 min (Figure 6(b)), and CLME also showed a peak at the same retention time, 60.629 min. Moreover, we also checked the content evaluation of (1R)-(-)-Myrtenal in the C. cernuum extract and the content values of (1R)-(-)-Myrtenal in the sample of CLME was 0.86 ± 0.15 mg/g (0.086 ± 0.015%). Therefore, this result suggested that CLME contained (1R)-(-)-Myrtenal as one of the major compounds. Next, we examined the effect of Myrtenal on viability of RAW 264.7 cells, and 1000 *μ*M of Myrtenal demonstrated obvious cytotoxicity (Figure 6(d)). Therefore, three concentrations (125, 250, and 500 *μ*M) of Myrtenal were chosen for the subsequent experiments. Similar to CLME, Myrtenal treatment (500 *μ*M) significantly (*P* < 0.001) suppressed LPS-induced production of NO, as well as the inflammatory cytokines TNF-*α* and IL-6, in a dose-dependent manner (Figures 6(e)–6(g)).

## 4. Discussion

Recent research has found various macrophages roles and functions, and macrophages play an important role in maintaining homeostasis and normal physiological conditions by coordinating a variety of biological activities [23]. Actually, studies examining the putative immunological properties of natural products and/or their derivatives depend heavily on the use of cell lines as an initial screen for biological activity [24]. In addition, because of their popularity as targeted single cells for assessing immune reactivity [25–28], we also chose RAW 264.7 macrophages for our current study. Macrophages can be stimulated by LPS to produce proinflammatory molecules (e.g., NO and PGE_2_) by activating intracellular signaling pathways, including NF-*κ*B and MAPK signaling pathways [29]. iNOS is the key enzyme responsible for the production of NO and is expressed excessively during an inflammatory response [8]. Accordingly, the production of these proinflammatory molecules is a major factor used to assess the efficacy of anti-inflammatory drugs.

NF-*κ*B-activating pathways are triggered by a variety of extracellular stimuli, leading to the phosphorylation and subsequent proteasome-mediated degradation of inhibitory molecules, the inhibitor of NF-*κ*B (I*κ*B) proteins [30], translocation of NF-*κ*B to the nucleus [31], and subsequent combination with their cognate DNA binding sites to modulate the transcription of more than 150 genes, such as proinflammatory cytokines, antimicrobial peptides, chemokines, antiapoptotic proteins, and stress-response proteins [32]. In this study, we noted that I*κ*B degradation and phosphorylation and NF-*κ*B translocation are effectively recovered by CLME in RAW 264.7 cells when triggered by LPS.

Chronic inflammation exerts its cellular side effects mainly through excessive production of free radicals and depletion of antioxidants [33]. Continued active inflammation response can lead to cell damage or cellular hyperplasia following ROS overproduction from inflammatory cells [33]. There is reversible interrelationship between oxidative stress and inflammation. The factors that cause inflammation and its amplification lead to oxidative stress and the reverse sequence of events (oxidative stress induced inflammation) [34]. ROS are the main oxidative products principally released by cytochrome p450 metabolism, peroxisomes, mitochondria, and inflammatory cell activation by endotoxins in macrophages [35]. ROS have been associated with numerous proinflammatory signal transduction cascades triggered by IL-1*β*, TNF-*α*, and LPS [36]. It was reported that ROS have the latent ability to activate NF-*κ*B in the early disease phase [37]. Here, HO-1 is important for protecting macrophages from ROS and has become an interesting target for antioxidant-related experiments [12]. In addition, its products, such as iron ions, CO, and biliverdin, modulate inflammation and exhibit critical effects against oxidative stress [38].

Nrf2 signaling constitutes one of the crucial cellular defense mechanisms in xenobiotic damage and oxidative stress [13]. Activation of Keap1/Nrf2 signaling manifests advantageous effects in a several disease states including diabetes, atherosclerosis, malaria, liver injury, obesity, neurodegenerative diseases, and certain cancers [39]. Under normal physiological conditions, Nrf2 is sequestrated in the cytoplasm and, upon stimulation, isolated from its cytosolic inhibitor Keap1, translocated into the nucleus, and then combined to the *cis*-acting antioxidant responsible element (ARE) in the promoter region [40]. The antioxidant features of ARE enzymes are involved in reducing the formation of adhesion molecules, such as NO and inhibition of oxidative stress [41]. It is also well known that there is crosstalk between NF-*κ*B and Nrf2, which regulates proinflammatory responses. Moreover, overexpression of Nrf2 can suppress the activation of the NF-*κ*B pathway. Furthermore, the elimination of Nrf2 exacerbated the activation of NF-*κ*B-induced inflammatory response [42]. Therefore, in this study, we examined whether CLME regulate the Keap1/Nrf2 signaling pathway, which is relevant to NO and ROS production, as well as the NF-*κ*B signaling. CLME effectively increased the nuclear accumulation of Nrf2 and concurrently downregulated the nuclear expression of NF-*κ*B in LPS-stimulated RAW 264.7 cells.

There are three well-defined subgroups of MAPKs in mammalian cells: ERK, JNK, and p38 MAPK. MAPKs are a chain of kinases connected with cell proliferation, cell survival, inflammation, apoptosis, tumorigenesis, and other physiological process [43]. Li et al. reported that the elevated ROS levels can activate a cascade of noxious events during inflammatory processes via MAPKs pathway, and ERK and p38 MAPK were reported to be strongly implicated in Nrf2 pathways [44]. Additionally, the phosphorylation of MAPKs in LPS-challenged macrophages can also stimulate the transcriptional activation of NF-*κ*B [29]. Our data demonstrated the phosphorylation of ERK, following LPS induction, was diminished by CLME treatment in the RAW 264.7 cells. Furthermore, CLME treatment also effectively alleviated the expression of p-MEK, upstream of p-ERK, compared to the LPS-treated group. The importance of ERK on inflammation, in particular, has been well reported. ERK is majorly correlated with LPS-induced macrophages inflammation [27, 45–48]. Additionally, it was reported that the suppression of ERK successfully inhibited inflammation in mouse and rabbit models [49, 50]. Therefore, ERK inhibitors can be effective targets for therapeutic intervention in inflammatory diseases. Collectively, these data suggested that inactivation of MEK/ERK expression could be involved in the action of CLME on the expression of NF-*κ*B and Keap1/Nrf2 pathway.

Excessive inflammation is the main cause of organ failure and mortality in sepsis [51], which is induced by pathogens like LPS-releasing Gram-negative bacteria [52]. CLME administration enhanced the survival rate in mice upon sepsis induction but not reached significance, indicating that CLME may slightly protect the mice from the septic shock. Although further investigations of animal experiment are still needed to prove that CLME has an anti-inflammatory effect *in vivo*, the present results support that CLME may inhibit the LPS-stimulated inflammation at least *in vitro*.

Previously, the principal constituents of essential oil of C. *cernuum* were identified as sesquicineole, *α*-bisabolol, and Myrtenal [19]. Among them, Myrtenal, a natural monoterpene, is one of the most abundant compounds in the Asteraceae family and is also a ubiquitous constituent of the essential oils of flowers, stems, and leaves [53]. In addition, Myrtenal is present in many medicinal plants such as pepper, cumin, mint, eucalyptus, and others [54]. Several studies have reported the various biological activities of Myrtenal, including antihepatocellular carcinoma activity in rats [55], antioxidant action against colon cancer in rats [56], and antihyperglycemic activity in diabetic rats [57]. Hari Babu et al. suggested that Myrtenal is being a good scavenger of NO, as well as a free radical inhibitor [58]. In addition, Myrtenal-treated rats showed significant decreases in ROS levels compared to the 1,2-dimethyl hydrazine- (DMH-) induced colon cancer rat model [56]. Similarly, we found that Myrtenal is one of the major compounds in CLME, and it significantly suppressed NO, TNF-*α*, and IL-6 production in LPS-stimulated RAW 264.7 cells, showing that our result is consistent with previous studies. Myrtenal, an active compound of CLME, may be associated with powerful effects of CLME against inflammation and oxidative stress in LPS-stimulated RAW 264.7 cells.

However, our study has some limitations. First, our experiment design did not allow us to examine in more depth the major compounds of CLME, although our data support a role of Myrtenal for anti-inflammatory effects. Second, we set the concentrations of Myrtenal *in vitro* higher than the content of that in CLME identified by HPLC. So we are going to do a further study for other major compounds of CLME and assessing Myrtenal with content in CLME on inflammatory responses. The inhibitory effects of CLME in LPS-stimulated RAW 264.7 cells could partially be involved in Myrtenal, which needs further investigation.

## 5. Conclusions

In our study, CLME revealed antioxidant/anti-inflammatory effects by regulating MEK/ERK, NF-*κ*B, and Keap1/Nrf2 signaling in LPS-stimulated RAW 264.7 macrophages, and these effects may be correlated with Myrtenal, one of the major compounds in CLME.

## Data Availability

The datasets used and/or analyzed in this study are available from the corresponding authors on reasonable request.

## Abbreviations

C. *cernuum*: *Carpesium cernuum* L
CLME: Methanolic extract of C. *cernuum*
ERK: Extracellular signal-related kinase
HO-1: Heme oxygenese-1
LPS: Lipopolysaccharide
MAPK: Mitogen-activating protein kinase
MEK: ERK kinase
NF-*κ*B: Nuclear translocation of nuclear factor-*κ*B
NO: Nitric oxide
Nrf2: Nuclear factor-erythroid 2 p45-related factor 2
Keap1: Kelch-like ECH-associated protein 1
IL-6: Interleukin-6
TNF-*α*: Tumor necrosis factor-*α*
PGE_2_: Prostaglandin E_2_
ROS: Reactive oxygen species.

## Supplementary Materials

Supplementary Materials Table 1: the time point of all markers. Fig. S1: effect of CLME on the viability of RAW 264.7 cells. Fig. S2: CLME increases the expression of HO-1 in LPS-stimulated RAW 264.7 cells.
