# Supplementary material for: Anti-Inflammatory and Antioxidant Effects of Carpesium cernuum L. Methanolic Extract in LPS-Stimulated RAW 264.7 Macrophages
Source: Mediators Inflamm. 2020 Aug 7;2020:3164239. doi: 10.1155/2020/3164239 (PMC7439783; doi:10.1155/2020/3164239)
Supplement: Supplementary Materials — Table 1: the time point of all markers. Fig. S1: effect of CLME on the viability of RAW 264.7 cells. Fig. S2: CLME increases the expression of HO-1 in LPS-stimulated RAW 264.7 cells. [file 3164239.f1.docx]

**Table 1. The time point of all markers.**

| Markers | Time point (LPS treatment) |
| --- | --- |
| NO | 48 hours |
| PGE_2_ | 24 hours |
| iNOS | 24 hours |
| p-IκB/IκB | 30 min |
| NF-κB | 30 min |
| Nrf2/Keap1 | 1 hours |
| MAPKs | 30 min |
| p-MEK | 20 min |
| TNF-α | 24 hours |
| IL-6 | 24 hours |

**Fig. S1. Effect of CLME on the viability of RAW 264.7 cells.**


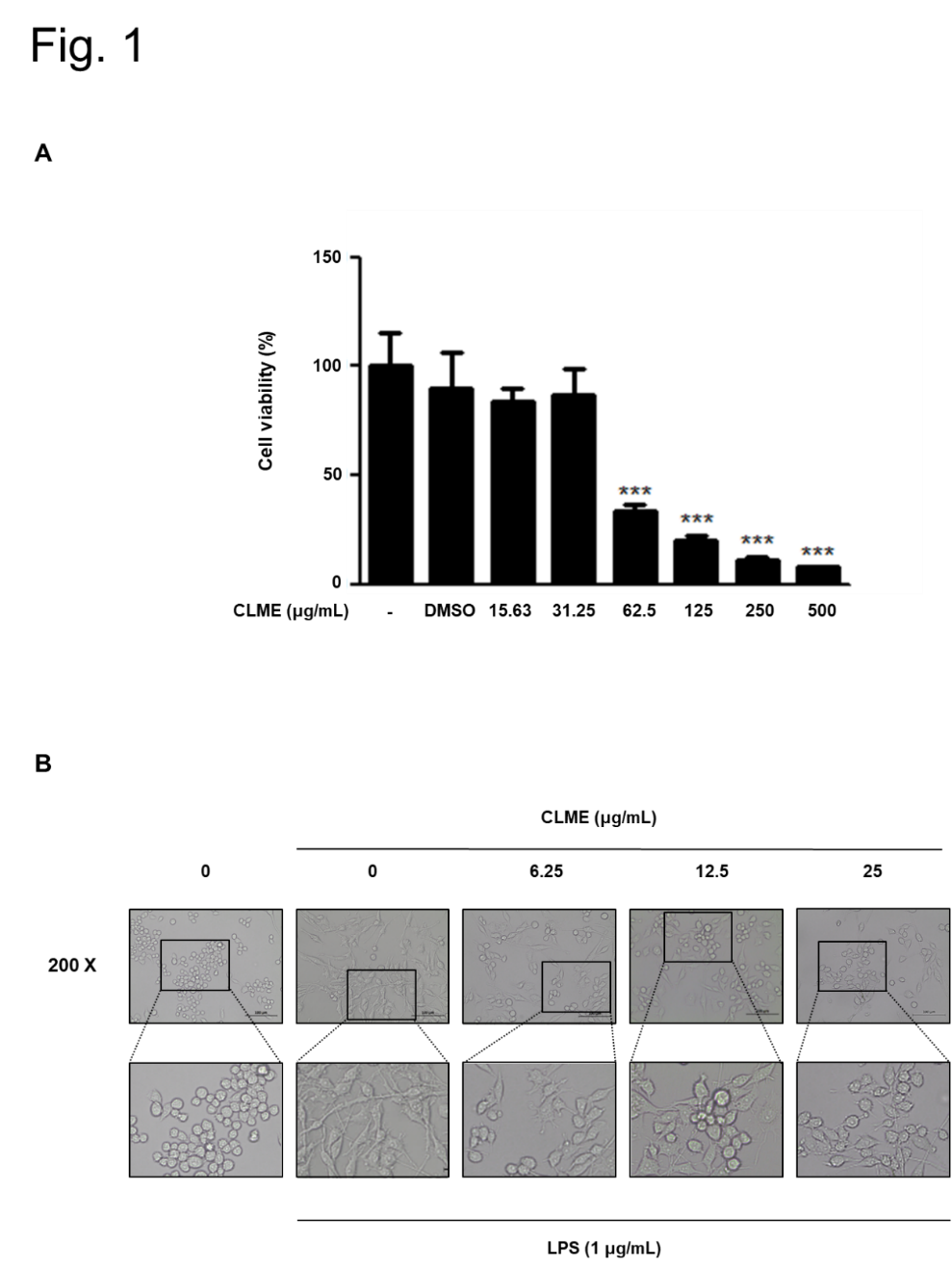
RAW 264.7 cells were treated with different concentrations of CLME for 24 h. (A) The viability of CLME in RAW 264.7 cells. (B) Microscopic analysis on morphology of cells. Images are shown at the original magnification of 200x. Scale bar is 100 µm. The values are represented as mean ± S.D. (n = 5). ^***^P < 0.001 vs. DMSO-treated group.

**Fig. S2. CLME increases the expression of HO-1 in LPS-stimulated RAW 264.7 cells.**

(A) The cells were treated with LPS of 1 μg/mL for the indicated times (B) The cells were treated with CLME of 25 μg/mL for 1h and followed by LPS stimulation (1 μg/mL) for indicated times. Densitometric analysis was performed using ImageJ ver. 1.50i. The values are represented as mean ± S.D. (n = 3). ^**^P < 0.01 and ^***^P < 0.001 vs. non-treated group.

**
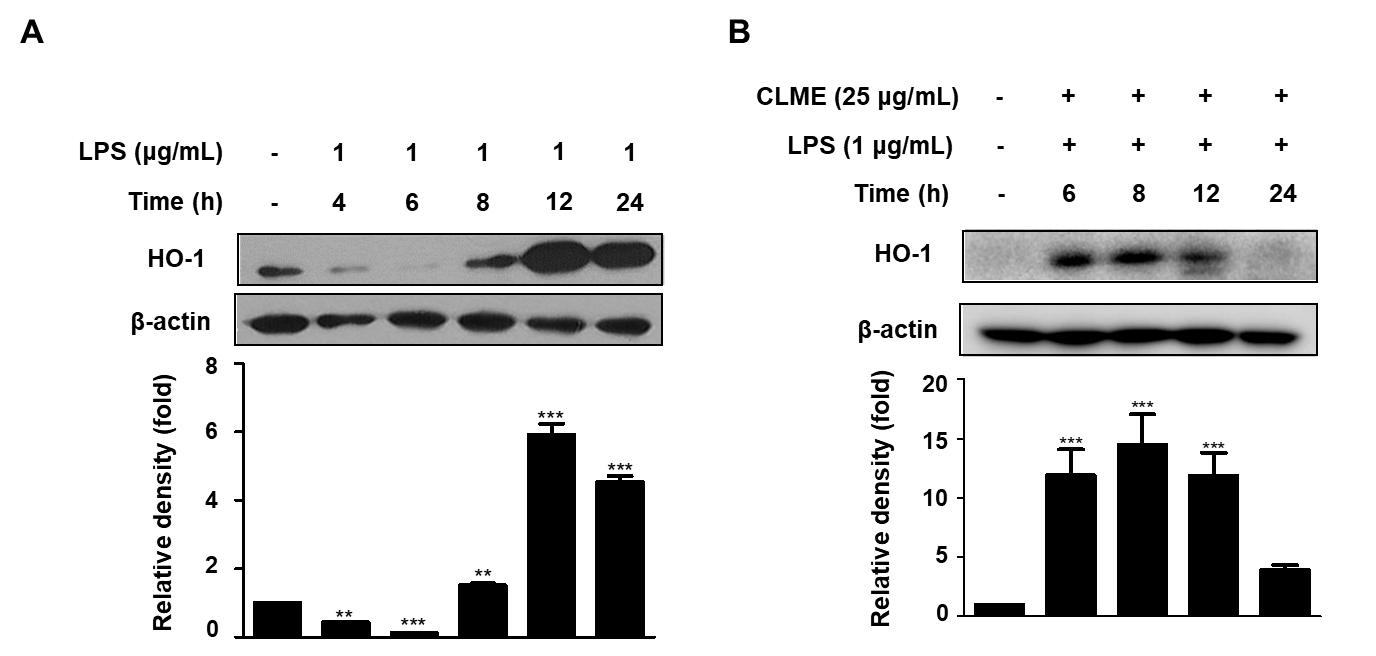
**
